# Potential Role of EPSPS Mutations in the Resistance of *Eleusine indica* to Glyphosate

**DOI:** 10.3390/ijms24098250

**Published:** 2023-05-04

**Authors:** Jingchao Chen, Hailan Cui, Zhiling Li, Haiyan Yu, Qiang Hou, Xiangju Li

**Affiliations:** 1 State Key Laboratory for Biology of Plant Diseases and Insect Pests, Institute of Plant Protection, Chinese Academy of Agricultural Sciences, Beijing 100193, China; 2 Institute of Biophysics, Chinese Academy of Sciences, Beijing 100101, China

**Keywords:** mutation, affinity, metabolomics, fitness, glyphosate, *Eleusine indica*

## Abstract

Gene mutation is a basic evolutionary mechanism in plants under selection pressure of herbicides. Such mutation has pleiotropic effects on plant growth. We systemically investigated the effects of Pro106Leu (P106L), Pro106Ser (P106S), and Thr102Ile + Pro106Ser (TIPS) mutations on EPSPS functionality and fitness traits in *Eleusine indica* at the biochemical and physiological levels. The affinity of natural EPSPS for glyphosate was 53.8 times higher than that for phosphoenolpyruvate (PEP), as revealed by the dissociation constant; the constant decreased in both the P106L (39.9-fold) and P106S (46.9-fold) mutants but increased in the TIPS (87.5-fold) mutant. The *Km* (PEP) values of the P106L, P106S, and TIPS mutants were 2.4-, 0.7-, and 4.1-fold higher than that of natural EPSPS, corresponding to resistance levels of 2.5, 1.9, and 11.4, respectively. The catalytic efficiency values (maximum reaction rates) were 0.89-, 0.94-, and 0.26-fold higher than that of natural EPSPS. The levels of metabolites related to amino acids and nucleotides were significantly reduced in the mutated plants. The fitness costs were substantial for the biomass, total leaf area, seed number, and seedling emergence throughout the growth period in the plants with P106L and TIPS mutations. These results provide insights into EPSPS kinetics and their effect on plant growth.

## 1. Introduction

The target of the global herbicide glyphosate 5-enolpyruvylshikimate-3-phosphate synthase (EPSPS; E.C. 2.5.1.19) catalyses the formation of aromatic amino acids and plays an essential role in the shikimate pathway [1,2]. Point mutations in the conserved regions of EPSPS, such as Pro 106, Thr 102, and Gly 101, are the mechanisms underlying the resistance of weed species [3,4,5,6,7]. Crystal structure analysis of recombinant protein of EPSPS in *Escherichia coli* has revealed structural changes in mutated EPSPS that are thought to prevent glyphosate binding, resulting in resistance, although the binding affinity is only indicated by *Km* values [5,8,9,10]. Changes in the active site also affect the EPSPS catalytic efficiency, which might result in fitness costs when treated using free glyphosate [11]. The fitness cost is a characteristic of herbicide-resistant weeds, where plants show a competitive disadvantage in the absence of herbicide selection pressure [11]. Seed production is reduced by at least 16% in a glyphosate-resistant (GR) population with a Pro106Ser (P106S) mutation in EPSPS compared with susceptible plants [12]. The fitness cost was significant (69% of the number of seeds produced) in a GR *Eleusine indica* population with a Thr102Ile + Pro106Ser (TIPS) double mutation in EPSPS [13]. The fitness cost of target site resistance (TSR) is not only associated with physiology, but also with genetic and biochemical factors that are triggered by a specific resistance gene under specific ecological backgrounds [11]. Estimating fitness costs is important for understanding the equilibrium frequencies of glyphosate resistance alleles in environments without herbicide selection and for developing a control strategy. However, few studies have systematically investigated the fitness traits of GR weed species at the genetic, biochemical, and physiological levels.

Goosegrass (Family: *Gramineae*; [*Eleusine indica* (L.) Gaertn.) is a widely-distributed annual weed found in temperate and tropical regions and can cause a 20%–50% reduction in the yield of infested crops, such as cotton (*Gossypium* spp.), watermelon [*Citrullus lanatus* (Thunb.) Matsum. and Nakai], and muskmelon (*Cucumis melo* L.) [14,15,16]. Several single-amino acid substitutions at Pro106 in EPSPS confer glyphosate resistance in *E. indica*, including Pro106Thr (P106T), Pro106Ala (P106A), Pro106Ser (P106S), and Pro106Leu (P106L) [17,18,19]. A double mutation (TIPS) in EPSPS has been identified in *E. indica* populations in Malaysia and China [20,21]. The present study investigated how different mutations in EPSPS of *E. indica* affect the kinetics, detectable dissociation constant (KD), and metabolite content, which affect the fitness and resistance levels. We expressed *E. indica* EPSPS with different mutations in *Escherichia coli* and measured the kinetic parameters, including the binding affinities for phosphoenolpyruvate (PEP) and glyphosate. We also generated homozygous GR *E. indica* populations with P106L, P106S, and TIPS mutations from similar backgrounds. We speculated that the *Km* (PEP) values only partially reflect the binding affinities between EPSPS and PEP, as the results showed an increased affinity of the mutated EPSPS to PEP with increased *Km* (PEP) values. This study provides a systematic approach to elucidating the fitness traits at the genetic, biochemical, and physiological levels.

## 2. Results

### 2.1. Analysis of Resistance to Glyphosate

**Figure 1:**
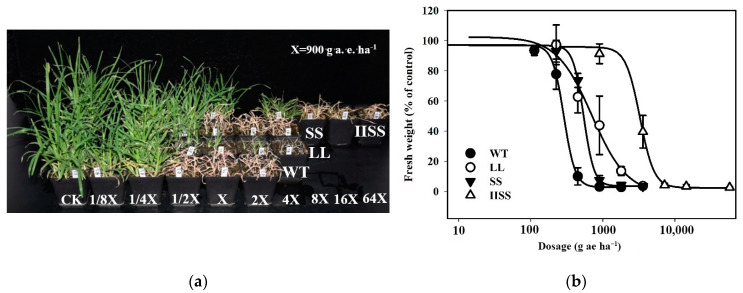

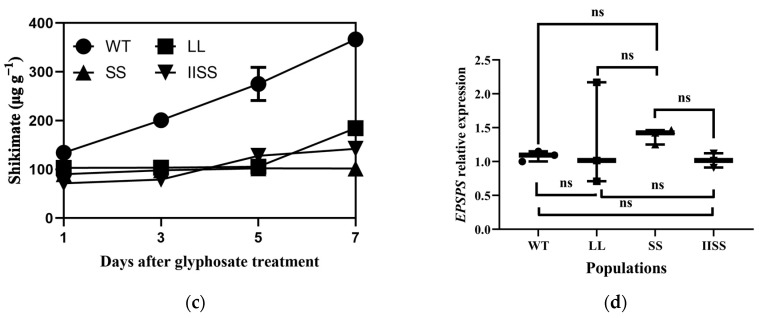
Responses of four *E. indica* populations (LL, SS, IISS, and WT) to glyphosate with different mutations (P106L, P106S, TIPS) in EPSPS. (**a**) Symptoms of injury to LL (homozygous mutation of P106L), SS (homozygous mutation of P106S), IISS (homozygous mutation of TIPS), and WT plants under different doses of glyphosate after 21 days. (**b**) Dose-responses curve of LL, SS, IISS, and WT *E. indica* plants to different doses of glyphosate calculated from fresh weight of aboveground tissues at 21 days after glyphosate treatment (DAT). Four pots were selected, and vertical bars represent standard errors of means (SEM). (**c**) Shikimate accumulation in leaf tissues of WT, SS, LL, and IISS populations at 1, 3, 5, 7 DAT at 1800 g a.e. ha^−1^. (**d**) Expression of *EPSPS* gene in leaf tissues of WT, SS, LL, and IISS populations that were not treated with glyphosate. Whole-plant assays, shikimate and *EPSPS* gene expression detection were repeated twice, and at least three biological replicates were designed. ns mean no significance.

The herbicide dose required for a 50% plant growth reduction (GR_50_) in the three *E. indica* populations with EPSPS mutations, calculated from the fresh weights at 21 days after treatment (DAT), ranged between 540.5 and 3272.9 g a.e. ha^−1^ (Figure 1a,b). The resistance indexes (RI) for the *E. indica* populations (IISS, LL, and SS) with homozygous mutations of TIPS, P106L, and P106S in EPSPS were 11.4, 2.5, and 1.9, respectively (Supplementary Table S1). This suggests that the resistance has evolved to a high level in IISS and to low levels in the LL and SS populations [20].

The glyphosate resistance indicator shikimate in wild type (WT) *E. indica* sharply increased under 1800 g a.e. ha^−1^ (7.1 mM) of glyphosate and reached 366.4 μg g^−1^ at seven DAT (Figure 1c). However, the shikimate accumulation in the IISS, LL, and SS plants was only 0.4-, 0.3-, and 0.5-fold, respectively, of that in the WT (Figure 1c). The low levels of shikimate in the tissues of these three populations indicated evolved resistance.

The expression levels of *EPSPS* in the WT, LL, SS, and IISS plants were similar (~1) relative to that of the reference gene acetolactate synthase (*ALS*; Figure 1d), indicating that *EPSPS* was not overexpressed in any of the four populations. In addition, the GR_50_ calculated from the fresh weights of the plants treated with glutathione S-transferase (GST)- and cytochrome P450 (P450)- inhibitors or glyphosate alone were similar (Supplementary Table S1). In addition, the growth was similar between the controls sprayed with water and the inhibitors. These results suggested that non-target resistance mechanisms were not responsible for the resistance to glyphosate in these four populations. 

### 2.2. EPSPS Kinetics Associated with Specific Mutations

**Figure 2:**
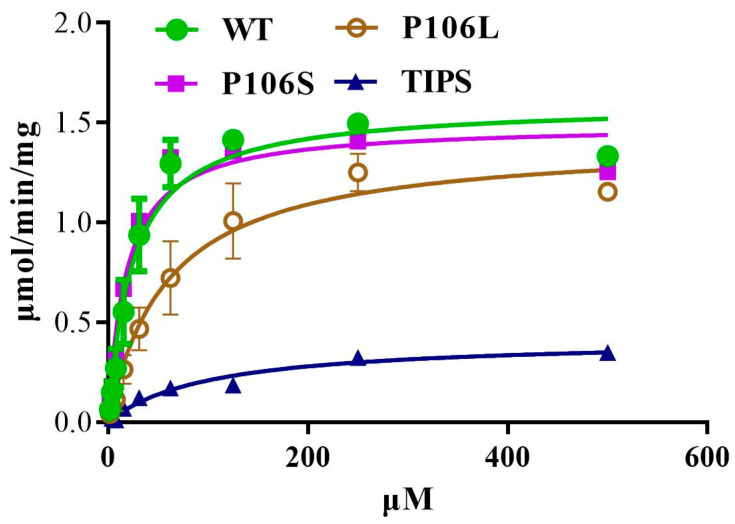
Characteristics of EPSPS with different mutations (WT, P106S, P106L, and TIPS) from purified enzyme extracts of *Escherichia coli* variants. Kinetic data of *Km* and *V_max_* of the His-tagged recombinant EiEPSPS variants for PEP were calculated using Michaelis–Menten equation. Reactions were detected as phosphate concentrations based on a standard curve (y = 97.049x − 0.4245, R^2^ = 0.99). This experiment was repeated twice with three biological replicates.

We expressed WT, LL, SS, and IISS *E. indica* 5-enolpyruvylshikimate-3-phosphate synthase (EiEPSPS) in *E. coli* and determined the *Km* and maximum reaction velocity (*V_max_*) of His-tagged recombinant EiEPSPS variants for PEP to characterise the mutations at the EPSPS level. The binding affinity of the four EPSPS enzymes to PEP and glyphosate was measured using a ForteBio Octet detection system. The IISS and LL mutations significantly increased the *Km* for PEP to 100.8 and 59.2 μmol min^−1^ mg^−1^, respectively (Table 1, Figure 2). However, the *Km* of the SS-mutated and WT were 18.2 and 24.6 μmol min^−1^ mg^−1^, respectively. The calculated maximum velocity (*V_max_*) of the reaction was also decreased for all three mutants (Table 1).

**Figure 3:**
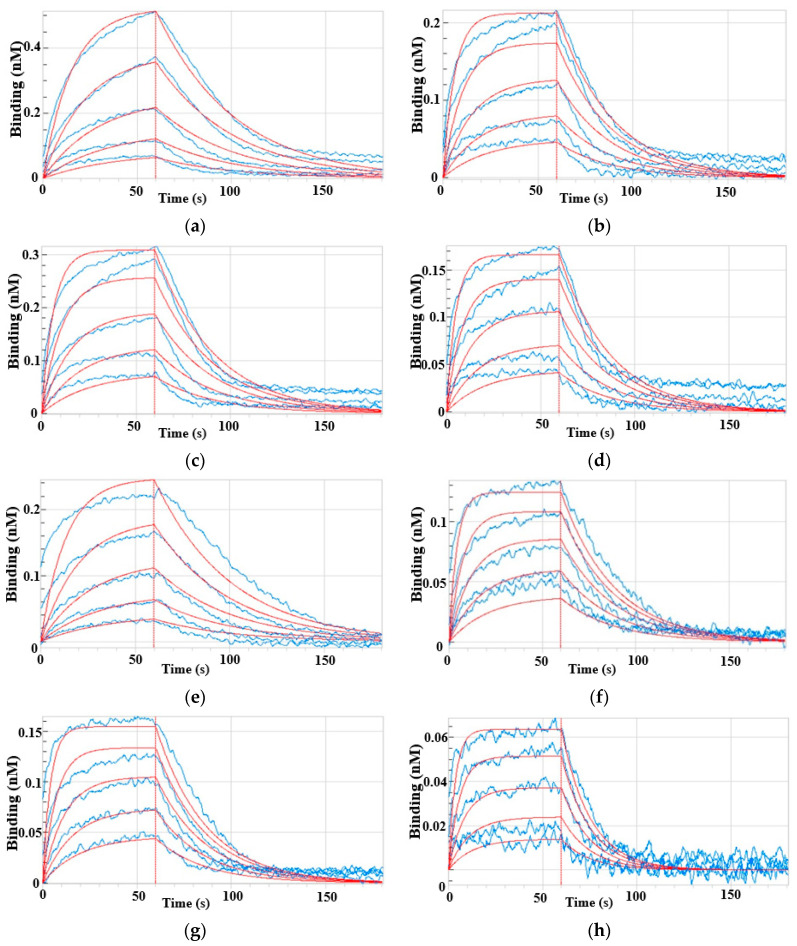
The kinetic analysis of protein-small molecule binding for EPSPS with different mutation by a ForteBio Octet 96 with high-capacity Super Streptavidin (SSA) biosensors. Biotinylated enzymes were loaded to the Super streptavidin sensors and then exposed to solutions containing serially diluted glyphosate (6.25, 12.5, 25, and 50 μM), and PEP (400, 800, 1600, 3200, and 6400 μM) for association (60 s), and to HEPES buffer containing 0.02% Tween 20 for dissociation (180 s). Association and dissociation steps are divided by the vertical red line. Sensorgrams are shown in blue. Fitting curves used for affinity calculation are in red. (**a**) WT to glyphosate; (**b**) P106L to glyphosate; (**c**) P106S to glyphosate; (**d**) TIPS to glyphosate; (**e**) WT to PEP; (**f**) P106L to PEP; (**g**) P106S to PEP; (**h**) TIPS to PEP. Two technical and three biological replicates were designed, respectively.

The binding affinity of the three mutant enzymes to PEP and glyphosate was measured using the ForteBio Octet system. The KD range of enzymes for glyphosate was 25.4–57.8 μM, compared with 1100.0–3100.0 μM for the natural substrate PEP (Figure 3, Table 1). The KD values indicate that the affinity of the enzymes was 39.9–87.5 fold higher for glyphosate than the natural substrate PEP, which agreed with the competitive enzyme binding mechanism through which glyphosate prevents weeds from growing. The KD values also revealed a 1.6- to 2.8-fold higher binding affinity for glyphosate or the natural substrate PEP in the mutant than natural EPSPS. Furthermore, the affinity of the P106L and P106S mutants was, respectively, 2.8- and 2.6-fold higher for PEP, and 2.1- and 2.3-fold higher for glyphosate compared with natural EPSPS. This indicated that these two enzymes tended to bind PEP more than glyphosate.

### 2.3. Fitness Traits of E. indica with Different EPSPS Mutations

**Figure 4:**
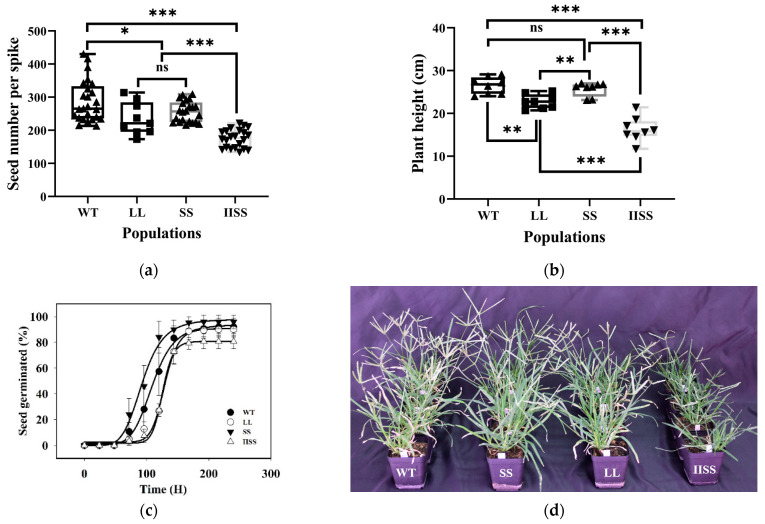
Fitness cost traits of *E. indica* with different mutations in EPSPS. (**a**) Seed number and (**b**) plant height per spike at mature stage of WT, SS, LL, and IISS populations. Values and vertical bars represent means (*n* = 24) and standard errors, respectively. *** *p* < 0.001; ** *p* < 0.01; * *p* < 0.05, ns mean no significance. (**c**) Germination results for IISS, LL, SS, and WT *E. indica* populations under day and night temperatures of 30 °C and 20 °C, respectively, and a 14-h photoperiod (*n* = 3). (**d**) IISS, LL, and SS, *E. indica* populations with mutations of Thr102Ile + Pro106Ser, Pro106Leu and Pro106Ser in EPSPS, respectively, and WT, wild type at reproductive growth stage. The fecundity traits were assessed in 24 plants from each population. Germination characteristics were designed three biological replicates for each population. This experiment was repeated twice.

The fitness traits, including the dry weight of the shoots and the total leaf area of the WT population, were significantly higher than those of the SS, LL, and IISS populations at most growth stages (Table 2). At 60 d after plant emergence, the dry weight was similar among the WT, SS, and LL populations in the second experiment. The trends for the dry weight and total leaf area were similar in SS and LL at all growth stages. However, these two physiological traits were significantly lower in the IISS than in the other populations. In addition, the seed yield in the WT population was much higher than that in the SS, LL, and IISS populations at 287, 235, 256, and 175 per plant, respectively (Figure 4a). Forty days after planting, the WT plants were much taller than the SS, LL, and IISS plants (26.5, 25.7, 22.8, and 16.3 cm, respectively) (Figure 4b,d). Under the selected conditions, the germination rates of the WT and SS populations were similarly maximal, and their T_50_ values were 109.9 and 93.4 h, respectively (Figure 4c). The T_50_ values were higher for the LL (129.1 h) and IISS (125.7 h) than for the WT population.

### 2.4. Metabolite Analysis

**Figure 5:**
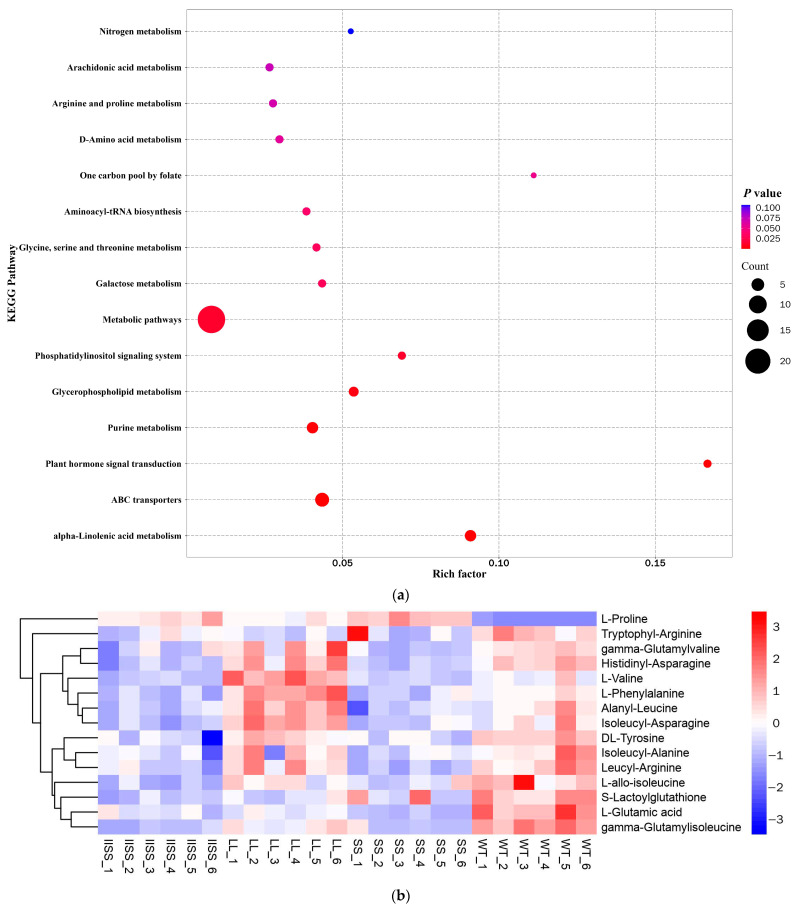

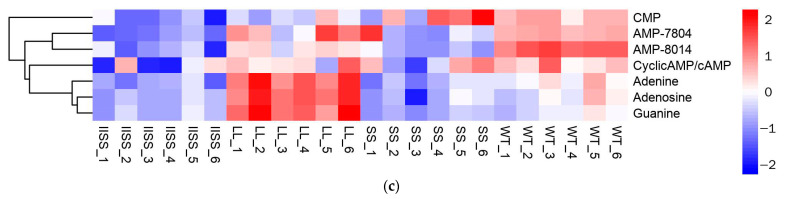
Differences in metabolite contents of four *E. indica* populations by determined by metabolomic analysis. (**a**) KEGG pathway enrichment bubble analysis of IISS vs. WT. Heat maps show classifications of metabolites associated with (**b**) carbon and (**c**) nitrogen metabolism in four *E. indica* populations. Two technical and six biological replicates was designed, respectively.

**Figure 6:**
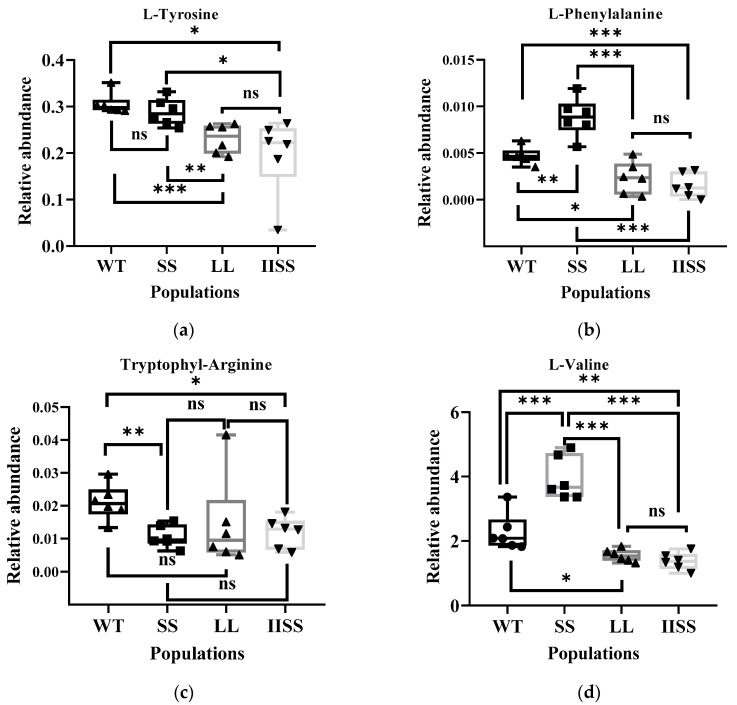

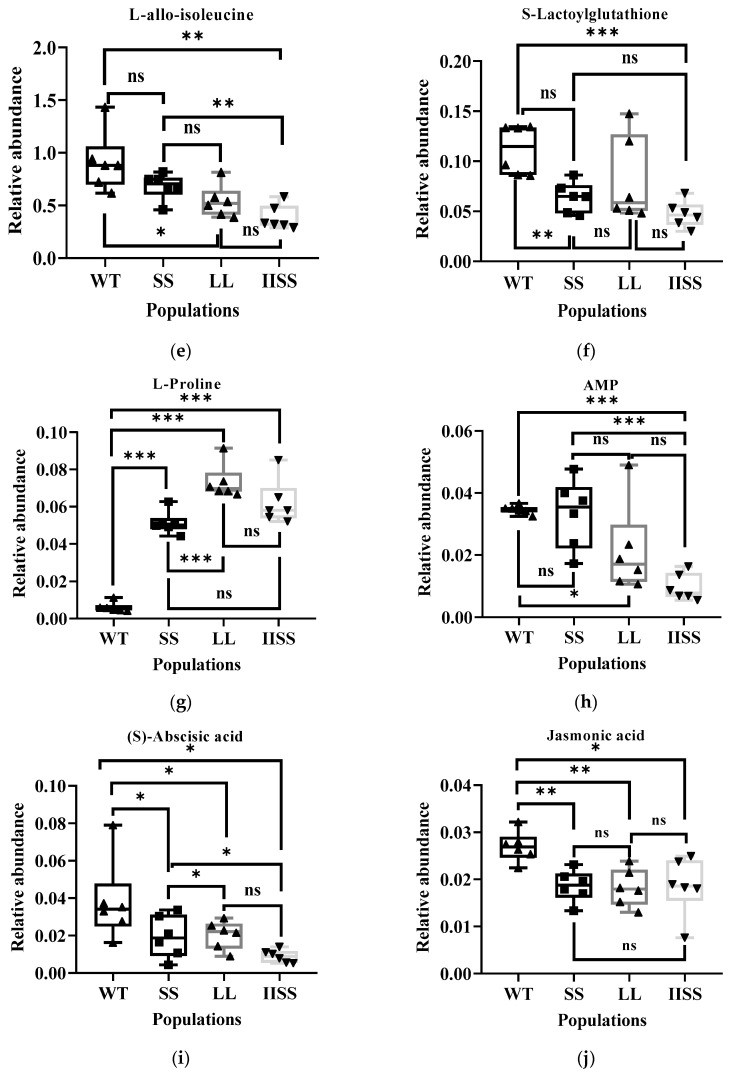
Differentially expressed metabolites associated with carbon and nitrogen metabolism among WT, SS, LL, and IISS populations. Values and vertical bars represent means and standard errors, respectively (*n* = 6). Significance levels are indicated by asterisks: *** *p* < 0.001; ** *p* < 0.01; * *p* < 0.05, ns mean no significance. (**a**) L-Tyrosine; (**b**) L-Phenylalanine, (**c**) Tryptophyl-Arginine, (**d**) L-Valine, (**e**) L-allo-isoleucine, (**f**) S-Lactoylglutathione, (**g**) L-Proline, (**h**) AMP, (**i**) (S)-Abscisic acid, (**j**) Jasmonic acid. Two technical and six biological replicates were designed, respectively.

The results of the principal component (PCA) and Orthogonal Projections to Latent Structures Discriminant (OPLS-DA) analyses revealed similar types and contents of metabolites among all of the samples in each group (Supplementary Figure S1). The range of values for *R^2^Y*, calculated using a permutation test, was 0.80–0.94 (Supplementary Figure S1). We found 223 downregulated and 58 upregulated metabolites in the IISS vs. WT group (Supplementary Table S2). The phenomenon was similar in the LL vs. WT groups, with more abundant downregulated than upregulated metabolites (Supplementary Table S2). However, 130 and 241 genes were, respectively, downregulated and upregulated in the SS vs. WT groups. Bubble analysis of the Kyoto Encyclopaedia of Genes and Genomes (KEGG) pathway enrichment indicated that the differentially expressed metabolites were included in the metabolic pathway (Figure 5a). The contents of metabolites associated with carbon and nitrogen metabolism were significantly higher in the WT than in the LL and IISS populations, whereas L-tyrosine, L-allo-isoleucine, and AMP were similar to those in the SS population (Figure 5b,c and Figure 6). However, the relative abundance of L-Proline was significantly lower in the WT than that in the three resistant populations (Figure 6). The contents of metabolites (S)-Abscisic acid and jasmonic acid, which are associated with plant hormones, were significantly higher in the WT than in the SS, LL, and IISS populations (Figure 6).

## 3. Discussion

Compared with the relatively low-level resistance in LL and SS, the IISS population showed high-level resistance to glyphosate, which was similar to previous research [20,22]. We also confirmed that the level of resistance endowed by the P106L mutation was higher than that of P106S in *E. indica*. This has been elucidated at the enzyme level only in *E. coli* [8,11]. In this study, as detected by ForteBio Octet, the binding affinity of glyphosate was 53.8 times higher for the natural EPSPS of *E. indica* than for PEP. These results indicated that glyphosate is more likely to bind to EPSPS and has a stronger competitive effect on the natural substrate, PEP [23,24]. In theory, glyphosate tolerance depends on the extent to which the inhibitor-binding site is perturbed, and the equivalent Gly96 in *E. coli* is critical for efficient glyphosate binding [25]. The present KD values revealed a higher affinity of both glyphosate and PEP for mutated, rather than natural, EPSPS. It is speculated that mutations in Pro106 and Thr102 might induce shifts in Gly101, which is the efficient binding site of glyphosate [25]. However, the ability of glyphosate and PEP to bind the enzyme is not significantly reduced because Gly101 is not mutated [9]. The *Km* of PEP, which is considered an indicator of the EPSPS binding affinity, remained unchanged in P106S, and slightly decreased in P106L [8,26,27]. The KD values of P106S and P106L were slightly decreased to 2.6- and 2.8-fold higher, respectively, compared with that of natural EPSPS. The *Km* for PEP in plants with the TIPS double mutation did not substantially change in *E. coli* (2.2-folds) [9] in a previous study (0.8-folds) [20], and in *E. indica* in the present study (4.4-folds). These values were similar to the 1.6-fold higher KD than those of WT in the present study. Structural modelling of EPSPS in *Tridax procumbens* has predicted that the Thr-102-Ser mutation weakly reduces glyphosate binding, but enhances PEP binding, and confers lower glyphosate resistance [28]. Above all, a structural analysis of EPSPS and the binding affinity determined here indicated that a single or double mutation in positions Thr102 and Pro106 alter the spatial structure, and shifts glyphosate away from the reaction center, but slightly affects the binding affinity of glyphosate for EPSPS and the natural substrate PEP. In addition, the *Km* (PEP) only partially reflected the binding affinity between EPSPS and PEP.

The resistant populations, particularly those with P106L and TIPS homozygous mutations in EPSPS, had fitness cost traits in the absence of glyphosate. Both the biomass and leaf area of the LL population were significantly lower than those of WT at all growth stages, and the number of seeds was 20% lower than that of WT. The present findings provide the first indication of fitness cost traits in a weed species with an evolved P106L mutation in EPSPS. The number of seeds was only 40% of that of WT in *E. indica* plants with the homozygous TIPS EPSPS variant. This was similar to a significant resistance cost of 50% of the seed number [13]. Only the population with the P106S mutation had a slight fitness cost during the early growth stage [13]. Amino acid substitutions in the conserved region of EPSPS endow the glyphosate resistance at the enzyme and plant levels, but alter the EPSPS catalytic efficiency, which is associated with the fitness trait. Here, the *V_max_* values of P106S and P106L were similar to that of natural EPSPS (0.94- and 0.89-fold, respectively). However, it was 0.26-fold higher than that of the natural EPSPS in TIPS. In addition, the increased *Km* values for P106L (2.4-fold) and TIPS (4.1-fold) indicated that the catalytic efficiency decreased. A P106L substitution compromises EPSPS *V_max_* by a factor of six in *E. coli* [8], but not in rice [26]. In addition, the P106L mutation reduces the EPSPS catalytic efficiency 5-fold in maize [27]. The activity of the single-site mutant enzyme P106S decreased by 2.5-fold, and the activity of EPSPS with a TIPS mutation decreased by nearly 9-fold [9]. 

Approximately 20% of the carbon fixed by plants flows through the shikimate pathway, which is associated with the synthesis of aromatic amino acids and secondary metabolites with diverse physiological roles [29]. An impaired catalytic capacity of EPSPS caused by point mutations could trigger several biochemical changes because this is a key enzyme in the shikimate pathway. Compared with the WT, the TIPS mutation resulted in 15.5- and 3.1-fold lower EPSPS activity (*V_max_*) here and in a previous study, respectively [20]. The decreased catalytic capacity of EPSPS affected the aromatic amino acids, which is consistent with previous studies [13]. However, shikimate and quinate are intermediate products in the shikimate pathway, but were not screened by UPLC-MS/MS in any of the four populations. The shikimate data accumulation during the initial part of this study revealed no differences among the four populations at one DAT following exposure to glyphosate (1800 g a.e. ha^−1^). These results indicate that the shikimate content might not be a key factor for fitness traits. The quinate accumulation after glyphosate treatment, plant growth, and net photosynthesis were confirmed to be affected by the exogenous quinate [30]. We previously found that some metabolites associated with auxin were increased in *E. indica* plants that overexpressed EPSPS and had fitness benefit traits [31]. However, these metabolites did not decrease in the three resistant populations with fitness cost traits. In addition, the decrease in the jasmonate in these three resistant populations indicated a low level of the secondary metabolite. The accumulation of proline in plant tissues is one characteristic of stressed plants. We found that 8.4–12.1-fold more Pro had accumulated in these three resistant populations than in the WT plants (Figure 6) [32]. The reduced content of metabolites relative to amino and nucleic acids indicates imbalanced carbon and nitrogen metabolism in the resistant populations.

## 4. Materials and Methods

### 4.1. Plant Materials and Whole-Plant Assay

Plants with homozygous mutations Pro106Ser (P106S), Pro106Leu (P106L), Thr102Ile + Pro106Ser (TIPS), and WT were screened from a population with a similar background, as we previously described [21,33]. Homozygosity and expression were confirmed through PCR and real-time quantitative PCR (qPCR), as described below (Supplementary Figure S2). Plants with the same confirmed genotype were cultured (bulk in isolation) and the seeds were produced and purified. The purified subpopulations were named SS, LL, IISS, and WT. Seeds with different EPSPS mutations were soaked in gibberellin (1 g·L^−1^) for 24 h to break dormancy, washed with sterile distilled water, and cultured in square plastic pots (8 × 8 cm) in a 3:1 (*v*/*v*) mixture of loam and organic fertiliser (organic content ≥ 15%). The plants were cultured under greenhouse conditions of day and night temperatures of 30 °C and 20 °C (±2 °C), respectively, with a relative humidity of 60% ± 20%, and a 14-h photoperiod, during which LEDs provided light intensity of 300 μmol m^−2^ s^−1^. After thinning, eight plants per pot were maintained [34]. When the plants reached the five- to seven-leaf stage, different doses of glyphosate (Supplementary Table S3) were applied using a moving TeeJet^®^ XR8002 flat fan nozzle cabinet sprayer (TeeJet Technologies, Glendale Heights, IL, USA) between 9–11 a.m. [18]. Thereafter, P450- (10 mM piperonylbutoxide) and GST-inhibitors (3 mM 4-chloro-7-nitro-1, 2, 3-benzoxadiazole) were sprayed to exclude other non-target-site resistance mechanisms. These inhibitors were also sprayed on another set of plants from these four populations at 3 and 72 h before glyphosate, respectively [31]. Water and inhibitors were applied through control spraying (Supplementary Tables S1 and S3). The fresh weight of the fresh aboveground tissues was measured at 21 DAT. Whole-plant assays were repeated twice in a completely randomised design.

### 4.2. Shikimate Accumulation Assays

Twice the recommended dose of glyphosate (~ 1800 g a.e. ha^−1^; 7.1 mM) was applied when the four populations (WT, LL, SS, and IISS) reached the 5–7-leaf stage. Leaf tissue samples collected on 1, 3, 5, and 7 DAT were finely ground in liquid nitrogen, added to 1.0 mL of 0.25 mol L^−1^ cold HCl, and centrifuged at 12,000 rpm for 30 min. Supernatants (0.2 mL) were incubated with 2 mL of 1% periodic acid for 3 h at room temperature. Thereafter, 2 mL of 1 mol L^−1^ NaOH and 1.2 mL of 0.1 mol L^−1^ glycine were added, then the optical density of 0.2 mL/well was determined in 96-well plates at 380 nm using a Tecan Infinite 200 Pro plate reader (Tecan Group Ltd., Männedorf, Switzerland). The results for all samples were compared with a standard curve of 0.1–400.0 μg shikimate and are expressed as μg of shikimate g^−1^ fresh tissue [18]. This experiment was repeated twice.

### 4.3. Sequencing and Expression of EPSPS

Genomic DNA was extracted using a Plant DNA extraction Kit (Tiangen Biotech, Beijing Co., Ltd., Beijing, China). Primers were designed to amplify a 310 bp fragment, which included the conserved region (Gly101 to Pro106) of *EPSPS* (Supplementary Table S4). Standard PCR proceeded with an annealing temperature set at 60 °C [21]. The PCR products were resolved by 1% agarose gel electrophoresis and sequenced (at Sangon Biotech Shanghai Co. Ltd., Beijing, China). Mutations were detected by aligning *EPSPS* sequences of all populations with a reference gene (KM387414.1) using DNAMAN software (Lynnon BioSoft, San Ramon, CA, USA).

The total RNA of 5–7-leaf tissues that were randomly collected from the population that was not sprayed with glyphosate was extracted using a plant RNA extraction kit (Tiangen Biotech Beijing Co., Ltd., Beijing, China). After confirming the quality of all samples through gel electrophoresis, first-strand complementary DNA was synthesised from 1 μg of RNA in a total volume of 20 μL using a kit (TransGen Biotech, Beijing, China). The qPCR reaction volume (20 µL) included primer pairs for EPSPS and the reference gene ALS (Supplementary Table S4) [35] and 2 × SYBR Green PCR master mix. The reaction proceeded on an ABI 7500 PCR system (Applied Biosystems, Foster City, CA, USA). The expression level of *EPSPS* relative to that of *ALS* was analysed using the 2^−△△Ct^ method [36]. This experiment of *EPSPS* gene expression detection was repeated twice.

### 4.4. EPSPS Protein Expression and Purification

Target *EPSPS* genes with different mutations were amplified through PCR using primers (Supplementary Table S4) and the synthesised cDNA, then cloned into the BamHI and HindIII cleavage sites of the pET-28a expression vector (New England Biolabs Inc., Ipswich, MA, USA). After DNA sequencing, the pET-28a plasmid (Thermo Fisher Scientific Inc., Waltham MA, USA) containing the target genes was transformed into BL21 (DE3) cells (Thermo Fisher Scientific Inc.). Selected clones were cultured in Luria-Bertani medium. Cells containing target genes that had grown to an OD_600_ of 0.5 were induced with 0.5 µM IPTG (0487; Amresco LLC, Solon, OH, USA) at 37 °C for 4 h. The cells were then pelleted through centrifugation at 10,000× *g* for 10 min and resuspended in 50 mM Tris-HCl and 150 mM NaCl, pH 7.4 (buffer A). The suspension was sonicated and centrifuged at 12,000× *g* for 30 min, the supernatant was filtered through a 0.22 µm membrane and passed through a HiTrap His column (10318950; Cytiva, Global Life Sciences Solutions USA, LLC., Marlborough, MA, USA). Target proteins were eluted with 50 mM Tris-HCl, 150 mM NaCl, 500 mM imidazole, pH 7.4 (buffer B), resolved through SDS-PAGE, and identified through MALDI-TOF mass spectrometry. Target protein concentrations were determined using the Bradford method (500-0001) [37] with Dye Reagent (both from Bio-Rad Laboratories Inc., Hercules, CA, USA). 

### 4.5. Biotinylation for EPSPS with Different Mutations

To prevent primary amines reducing the biotinylation ratio, the EPSPS and mutations were exchanged with PBS buffer using a PD10 desalting column (17085101; Cytiva, Global Life Sciences Solutions USA, LLC.) as described by the manufacturer. The proteins were then concentrated using an ultrafilter (Merck KGaA, Darmstadt, Germany), then the concentrations were determined, as described above. The proteins were biotinylated using the EZ-Link™ Sulfo-NHS-LC-biotinylation kit (21435; Thermo Fisher Scientific Inc.) as described by the manufacturer. A mixture of protein and sulfo-NHS-LC-biotin at a molar ratio of 1:5 was incubated at 4 °C for 16 h, then free biotin molecules were removed using a PD10 desalting column and biotinylated protein concentrations were measured using the Bradford method.

### 4.6. Kinetic Characterisation Assay

We used a ForteBio Octet 96 with high-capacity Super Streptavidin (SSA) biosensors (18-5057; Sartorius Stedim Biotech GmbH, Göttingen, Germany) to study the protein-small molecule binding, and to derive the relevant kinetic data, as well as the affinity constants [38]. The SSA sensors were activated in 50 mM HEPES buffer (pH 7.5) containing 0.02% Tween 20 (*v*/*v*) for 10 min, then biotinylated proteins were loaded onto them online for 2 min. Thereafter, the sensors were quenched with 2 μg mL^−1^ of biocytin (B1592 Thermo Fisher Scientific Inc.) for 60 s. Kinetics of the association and disassociation of serial dilutions of PEP or glyphosate and proteins were assayed at baseline for 60 and 180 s, respectively, in HEPES buffer (pH 7.5) containing 0.02% Tween 20 (*v*/*v*). To obtain accurate interaction kinetics, a reference well (containing only buffer) and a reference biosensor were included in the assay for double subtraction analysis.

### 4.7. Fitness Traits Assay without Competition

Fitness traits were assessed in the absence of glyphosate or competition. Seeds from all populations were cultured under the conditions described above, and only one individual was maintained in each pot. The total leaf area for all aboveground parts was determined using a YMJ-B leaf area meter, Zhejiang Top Cloud-agri Technology Co., Ltd., Zhuji City, China) three times every 20 d after the plants emerged, and the biomass was dried at 80 °C for 48 h and weighed [39]. The fecundity traits of mature seed numbers were assessed in 24 plants from each population. Germination characteristics were analysed for four population in 9 × 9-cm^2^ Petri dishes; 30 seeds were placed in each Petri dish, lined with filter paper and moistened with 5 mL distilled water. The dishes were placed in an RXZ-310D artificial climate chamber (Jiangnan Instrument, Ningbo, China) under a 16-h photoperiod, 21 μmol·m^−2^·s^−1^ light intensity, and day and night temperatures of 30 °C and 20 °C, respectively. Germinated seeds (radicle length > 2 mm) were counted and removed from the Petri dishes, then 1 mL of distilled water was added daily for 10 d [40].

### 4.8. Metabolite Analysis

Leaf tissues from six randomly selected individuals per population were selected and quickly frozen in liquid nitrogen, then the metabolites were analysed (Baiqu Biomedical Technology Co., Ltd., Shanghai, China). Briefly, tissues (50 mg per sample) in 1 mL of methanol:water = 3:1 (isotopically labelled internal standard mixture; extraction solution) were homogenised at 35 Hz for 4 min then sonicated for 5 min in an ice-water bath three times. The samples were incubated for 1 h at −4 °C, then centrifuged at 13,800× *g* (R = 8.6 cm) for 15 min at 4 °C. The supernatants, transferred into fresh glass vials, were further analysed. A quality control (QC) sample comprised equal portions of all supernatants. 

The supernatants were analysed through LC-MS/MS using a Vanquish UHPLC system (Thermo Fisher Scientific Inc.) with a UPLC HSS T3 column (2.1 × 100 mm, 1.8 μm) coupled to an Orbitrap Exploris 120 mass spectrometer (Thermo Fisher Scientific Inc.). The mobile phase consisted of 5 mM ammonium acetate and 5 mM acetic acid in water (A) and acetonitrile (B), respectively. The auto-sampler temperature was 4 °C and the injection volume was 2 μL. We used an Orbitrap Exploris 120 mass spectrometer to acquire MS/MS spectra in an information-dependent acquisition (IDA) mode under the control of Xcalibur acquisition software (Thermo Fisher Scientific Inc.), which continuously evaluated the full-scan MS spectra. The ESI source conditions were as follows: sheath gas flow rate, as 50 Arb; Aux gas flow rate, 15 Arb; capillary temperature, 320 °C; full MS resolution, 60,000, MS/MS resolution, 15,000; collision energy, 10/30/60 in NCE mode; spray voltage, 3.8 kV (positive) or −3.4 kV (negative) [41]. 

The raw metabolite data were converted to the mzXML format using ProteoWizard and processed with an in-house program designed by Baiqu Biomedical Technology (Shanghai, China), and developed using R and based on XCMS, for peak detection, extraction, alignment, and integration. Metabolites were annotated using an in-house MS2 database (BiotreeDB) with a cut-off of 0.3 [42].

### 4.9. Statistical Analyses

Data from the whole-plant assays and emerged seedlings were analysed through nonlinear regression using a log-logistic model: *y* = *y*_0_ + *a*/[1 + (*x*/*x*_0_)*^b^*] in SigmaPlot 12.0 (Systat Software, San Jose, CA, USA) [43,44]. Where *y* is the inhibition rate, *x* is the glyphosate dose (g a.e. ha^−1^), *b* is the curve slope around *x*_0_, *y*_0_ is the lower limit, *a* is the difference between the upper and lower limits, and *x*_0_ is the herbicide dose required for GR_50_, or the time required for 50% of the seeds to germinate (T_50_). The RI is calculated as the ratio of GR_50_ of the resistant to that of the WT population [33]. 

Data for protein-small molecule binding studies and curve fitting were analysed using ForteBio Data Analysis Software v. 9.0. Dissociation constants (KD) indicating binding affinity for protein-small molecules were derived by determining K_on_ and K_off_ (association and disassociation rate constants, respectively); KD = K_off_/K_on_ [38].

We used SIMCA v. 16.0.2 (Sartorius Stedim Data Biotech GmbH) for the PCA and OPLS-DA of the metabolites. Differentially expressed metabolites were screened based on variable importance in projection (VIP) values > 1 and a false discovery rate < 0.05 [31]. 

The results of the *EPSPS* expression and fitness traits among populations were analysed using Student *t*-tests. All data were statistically analysed using SPSS 13 (SPSS Inc., Chicago, IL, USA).

## 5. Conclusions

Our research demonstrated the function of EPSPS with different mutations and the effects of such mutations on *E. indica* plant growth at the biochemical and physiological levels. The affinity of the herbicide glyphosate was higher for EPSPS with and without mutation than for its natural substrate PEP. We hypothesise that the *Km* of PEP only partially reflects the binding affinities with EPSPS because of the increased affinity of the mutated EPSPS for PEP. The catalytic efficiency of the mutated EPSPS decreased, resulting in disordered carbon and nitrogen metabolism in the plants. Moreover, the fitness cost traits were detectable and significant in the plants with P106L and TIPS mutations, and the numbers of seeds in such plants was 20–60% lower than that in the WT plans. The present results are important for designing resistance-management practices that can minimise the evolution of glyphosate resistance in *E. indica*.

## Figures and Tables

**Table 1:** Activity of EPSPS activity, *Km* (PEP), and binding affinity for glyphosate inhibitor and natural PEP substrate of mutated EPSPS expression in *E. coli*.

Genotype	EPSPS *V_max_*	*Km* (PEP)	K_on_ (M^−1^ S^−1^) ^1^		K_off_ (S^−1^) ^2^		KD (M) ^3^		
			Glyphosate	PEP	Glyphosate	PEP	Glyphosate	PEP	Ratio
Wild type	1.6	24.6	417.6	8.0	2.4 × 10^−2^	2.5 × 10^−2^	5.8 × 10^−5^	3.1 × 10^−3^	53.8
	(0.1)	(5.0)	(9.0)	(0.3)	(2.0 × 10^−4^)	(3.6 × 10^−4^)	(1.3 × 10^−6^)	(1.1 × 10^−4^)	
Pro106Ser	1.5	18.2	1264.0	31.8	3.2 × 10^−2^	3.8 × 10^−2^	2.5 × 10^−5^	1.2 × 10^−3^	46.9
	(0.1)	(2.8)	(31.1)	(0.7)	(4.2 × 10^−4^)	(4.8 × 10^−4^)	(7.1 × 10^−7^)	(3.0 × 10^−5^)	
Pro106Leu	1.41	59.2 (13.8)	1254.0	31.3	3.5 × 10^−2^	3.5 × 10^−2^	2.8 × 10^−5^	1.1 × 10^−3^	39.9
	(0.1)		(30.3)	(0.7)	(4.6 × 10^−4^)	(4.3 × 10^−4^)	(7.2 × 10^−7^)	(2.8 × 10^−5^)	
Thr102Ile + Pro106Ser	0.4	100.8 (18.1)	1647.0	32.95	3.7 × 10^−2^	6.5 × 10^−2^	2.3 × 10^−5^	2.0 × 10^−3^	87.5
	(0.3 × 10^−1^)		(52.8)	(1.3)	(6.7 × 10^−4^)	(1.5× 10^−3^)	(8.3 × 10^−7^)	(8.9× 10^−5^)	

^1^ K_on_, association rate constant; ^2^ K_off_, disassociation rate constant; ^3^ KD, dissociation constant derived by determining, KD = K_off_/K_on_.

**Table 2:** Dry biomass and total leaf area of individual plants from IISS, LL, SS, and WT populations collected at 20, 40, and 60 days after planting.

Repeat	Population	Dry Weight (mg Plant^−1^)			Leaf Area (cm^2^ Plant^−1^)		
		20 d	40 d	60 d	20 d	40 d	60 d
Experiment 1	WT	0.48 ^a^ *	1.55 ^a^	3.28 ^a^	98.75 ^a^	263.63 ^a^	314.38 ^a^
	LL	0.32 ^b^	1.46 ^b^	2.37 ^bc^	64.60 ^b^	245.98 ^b^	250.29 ^b^
	SS	0.27 ^b^	1.25 ^b^	2.67 ^b^	61.55 ^b^	233.66 ^b^	289.62 ^ab^
	IISS	0.12 ^c^	0.84 ^b^	2.00 ^c^	28.80 ^c^	159.58 ^b^	256.83 ^b^
Experiment 2	WT	0.08 ^a^	0.47 ^a^	1.92 ^a^	25.22 ^a^	130.44 ^a^	167.78 ^a^
	LL	0.03 ^c^	0.36 ^b^	1.65 ^a^	13.93 ^b^	92.78 ^c^	111.63 ^b^
	SS	0.05 ^b^	0.42 ^ab^	1.72 ^a^	26.08 ^a^	109.17 ^b^	116.38 ^b^
	IISS	0.03 ^c^	0.22 ^c^	1.32 ^b^	15.09 ^b^	62.85 ^d^	90.88 ^c^

* Superscript letters indicate significant differences among mean values of the four populations for each experiment (Tukey tests at 5% probability). IISS, LL, and SS, *E. indica* population with Thr102Ile + Pro106Ser, Pro106Leu and Pro106Ser mutations in EPSPS, respectively; WT, wild-type.

## Data Availability

Not applicable.

## Supplementary Materials

The following supporting information can be downloaded at: https://www.mdpi.com/article/10.3390/ijms24098250/s1, Figure S1. Differential metabolites analysis of individuals from four populations; Figure S2. Partial nucleotide sequence of EPSPS in four populations; Table S1. Parameter (SE) of non-linear model used for whole-plant assays of non-target-site resistance mechanisms detection.; Table S2. Number of differentially expressed metabolites among groups; Table S3. Dose designed for dose-response assay; Table S4. Primers were used in this study.
