# Supplementary material for: Potential Role of EPSPS Mutations in the Resistance of Eleusine indica to Glyphosate
Source: Int J Mol Sci. 2023 May 4;24(9):8250. doi: 10.3390/ijms24098250 (PMC10179075; doi:10.3390/ijms24098250)
Supplement: Supplementary file 1 [file ijms-24-08250-s001.zip › ijms-2367490-supplementary/Supplementary Table S1.pdf]

Supplementary Table S1. Parameter (SE) of non-linear model used for whole-plant assays of non-target-site resistance mechanisms detection.

Population	*b*	y_0	*a*	x_0	R^2	*P*	*RI*
WT	5.5 ± 0.7	2.7 ± 1.8	94.4 ± 2.7	287.6 ± 11.2	0.99	<0.0001	-
LL	1.8 ± 0.5	-1.8 ± 9.6	104.3 ± 12.2	707.2 ± 122.1	0.92	<0.0001	2.5
SS	6.0 ± 1.3	3.6 ± 2.1	93.2 ± 3.1	540.5 ± 25.6	0.98	<0.0001	1.9
IISS	4.4 ± 1.9	2.4 ± 2.1	93.4 ± 3.2	3272.9 ± 164.1	0.98	<0.0001	11.4
WT*	3.8 ± 0.5	12.1 ± 2.3	88.6 ± 3.4	309.7 ± 15.0	0.99	<0.0001	-
LL*	1.5 ± 0.2	4.7 ± 6.0	98.0 ± 7.0	854.3 ± 94.6	0.98	<0.0001	2.8
SS*	3.0 ± 0.3	5.1 ± 2.0	92.5 ± 3.0	606.5 ± 24.9	0.99	<0.0001	2.0
IISS*	1.6 ± 0.4	12.5 ± 4.9	91.0 ± 7.0	3829.6 ± 530.2	0.96	<0.0001	12.4

* Individuals treated by the GST- and P450- inhibitors. The non-linear model is $y=y_0+a/[1+(x/x_0)^b]$, In this model, *y* is the inhibition rate, *x* is the glyphosate dose (g a.e. ha^{-1}), *b* is the curve slope around x_0, y_0 is the lower limit, *a* is the difference between the upper and lower limits, and x_0 is the herbicide dose required for 50% plant growth reduction (GR_{50}). WT: Wild type, IISS: *E. indica* population with mutation of Thr102Ile + Pro106Ser in EPSPS; LL: *E. indica* population with mutation of Pro106Leu in EPSPS; SS: *E. indica* population with mutation of Pro106Ser in EPSPS.
