# Supplementary material for: Potential Role of EPSPS Mutations in the Resistance of Eleusine indica to Glyphosate
Source: Int J Mol Sci. 2023 May 4;24(9):8250. doi: 10.3390/ijms24098250 (PMC10179075; doi:10.3390/ijms24098250)
Supplement: Supplementary file 1 [file ijms-24-08250-s001.zip › ijms-2367490-supplementary/Supplementary Table S2.pdf]

Supplementary Table S2. Number of differentially expressed metabolites among groups.

Treatment groups	Differentially metabolites	Down-regulated	Up-regulated
IISS vs WT	281	223	58
SS vs WT	371	130	241
LL vs WT	309	225	84

Note: WT: Wild type, IISS: *E. indica* population with mutation of Thr102Ile + Pro106Ser in EPSPS; LL: *E. indica* population with mutation of Pro106Leu in EPSPS; SS: *E. indica* population with mutation of Pro106Ser in EPSPS. Differential metabolites were screened based on variable importance in projection (VIP) values > 1 and a false discovery rate < 0.05.
