# Supplementary material for: Potential Role of EPSPS Mutations in the Resistance of Eleusine indica to Glyphosate
Source: Int J Mol Sci. 2023 May 4;24(9):8250. doi: 10.3390/ijms24098250 (PMC10179075; doi:10.3390/ijms24098250)
Supplement: Supplementary file 1 [file ijms-24-08250-s001.zip › ijms-2367490-supplementary/Supplementary Table S4.pdf]

Supplementary Table S4. Primers used in this study.

Gene	Foward primer (5′-3′)	Reverse primer (5′-3)
EPSPS clone	5′-cgcggatccCACACTCTCTCTCTCTC-3′	5′-cccaagcttTTAGTTCTTGACGAAAGTGC-3′
qPCR for *EPSPS* gene	GCAATTTCCCCAGTGACGACC	GCAAAAGCCTCTATCTTCCCTGT
Reference gene (*ALS*)	GGTGGCAAGGTTAAGTTATCTGG	TCAACATAAGGGATGGAGATCAG
