# Supplementary material for: Potential Role of EPSPS Mutations in the Resistance of Eleusine indica to Glyphosate
Source: Int J Mol Sci. 2023 May 4;24(9):8250. doi: 10.3390/ijms24098250 (PMC10179075; doi:10.3390/ijms24098250)
Supplement: Supplementary file 1 [file ijms-24-08250-s001.zip › Supplementary files/Supplementary Figure S1.docx]

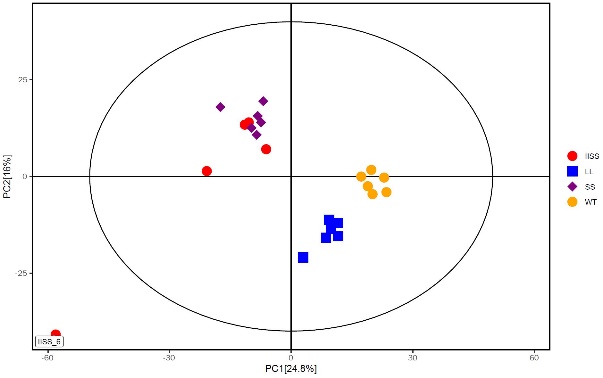

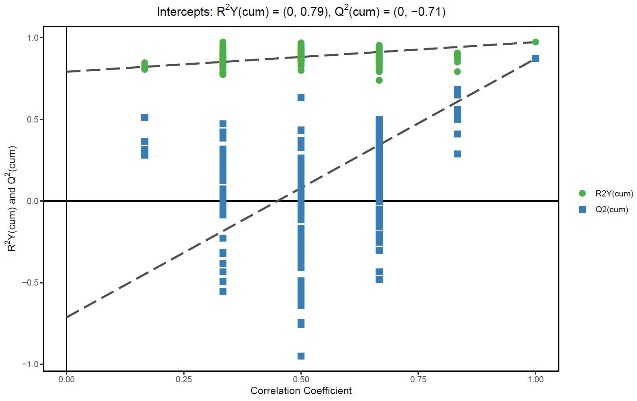


B

A


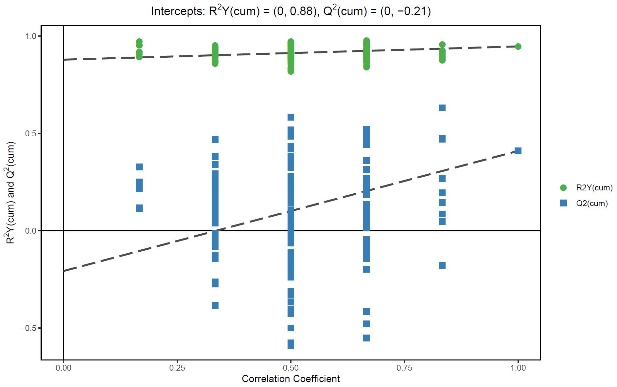

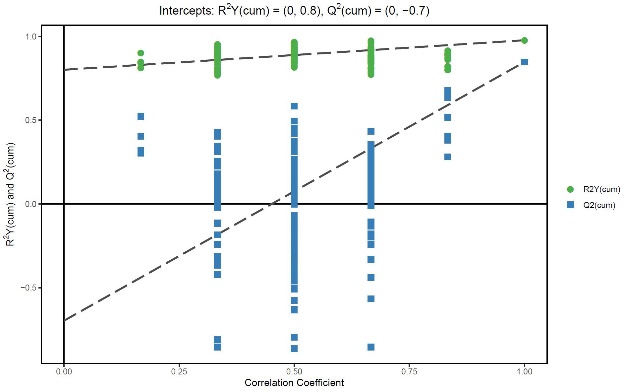


D

C


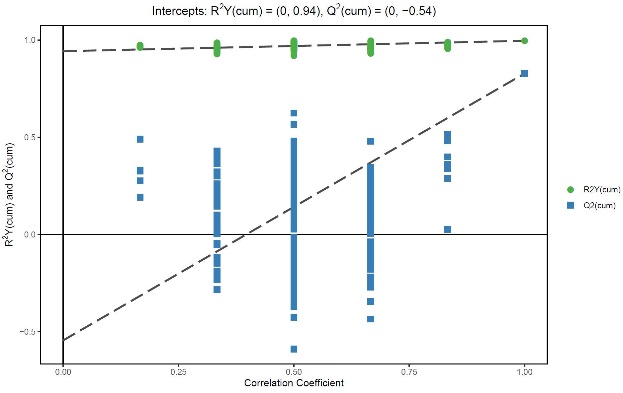

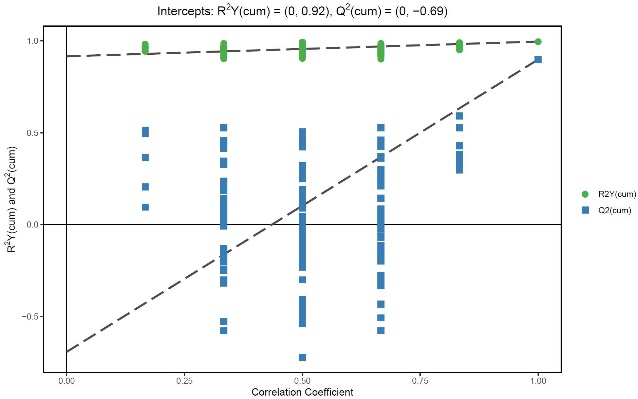


F

E


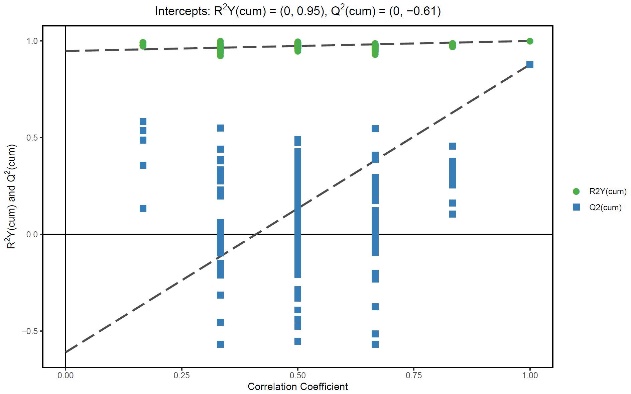


G

**Supplementary Figure S1.** Differential metabolites analysis of individuals from four populations. A, Score plot of four population from PCA model. B, Permutation plot for group of IISS-LL from OPLS-DA model; C, Permutation plot for group of IISS-SS from OPLS-DA model; D, Permutation plot for group of IISS-WT from OPLS-DA model; E, Permutation plot for group of LL-WT from OPLS-DA model; F, Permutation plot for group of SS-LL from OPLS-DA model; G, Permutation plot for group of SS-WT from OPLS-DA model; Correlation coefficient as the x-axis represents the replacement reservation degree of replacement test, and the y-axis represents the value of R^2^Y (green dots) and Q^2^ (blue square dots). The two dashes represent the regression lines of R^2^Y and Q^2^, respectively.
