# Supplementary material for: Potential Role of EPSPS Mutations in the Resistance of Eleusine indica to Glyphosate
Source: Int J Mol Sci. 2023 May 4;24(9):8250. doi: 10.3390/ijms24098250 (PMC10179075; doi:10.3390/ijms24098250)
Supplement: Supplementary file 1 [file ijms-24-08250-s001.zip › Supplementary files/Supplementary Table S1.docx]

**Supplementary Table S1.** Parameter (SE) of non-linear model used for whole-plant assays of non-target-site resistance mechanisms detection.

| Population | *b* | *y_0_* | *a* | *x_0_* | *R^2^* | *P* | *RI* |
| --- | --- | --- | --- | --- | --- | --- | --- |
| WT | 5.5 ± 0.7 | 2.7 ± 1.8 | 94.4 ± 2.7 | 287.6 ± 11.2 | 0.99 | <0.0001 | - |
| LL | 1.8 ± 0.5 | -1.8 ± 9.6 | 104.3 ± 12.2 | 707.2 ± 122.1 | 0.92 | <0.0001 | 2.5 |
| SS | 6.0 ± 1.3 | 3.6 ± 2.1 | 93.2 ± 3.1 | 540.5 ± 25.6 | 0.98 | <0.0001 | 1.9 |
| IISS | 4.4 ± 1.9 | 2.4 ± 2.1 | 93.4 ± 3.2 | 3272.9 ± 164.1 | 0.98 | <0.0001 | 11.4 |
| WT* | 3.8 ± 0.5 | 12.1 ± 2.3 | 88.6 ± 3.4 | 309.7 ± 15.0 | 0.99 | <0.0001 | - |
| LL* | 1.5 ± 0.2 | 4.7 ± 6.0 | 98.0 ± 7.0 | 854.3 ± 94.6 | 0.98 | <0.0001 | 2.8 |
| SS* | 3.0 ± 0.3 | 5.1 ± 2.0 | 92.5 ± 3.0 | 606.5 ± 24.9 | 0.99 | <0.0001 | 2.0 |
| IISS* | 1.6 ± 0.4 | 12.5 ± 4.9 | 91.0 ± 7.0 | 3829.6 ± 530.2 | 0.96 | <0.0001 | 12.4 |

* Individuals treated by the GST- and P450- inhibitors. The non-linear model is *y*=*y_0_*+*a*/[1+(*x*/*x_0_*)*^b^*], In this model, *y* is the inhibition rate, *x* is the glyphosate dose (g a.e. ha^-1^), *b* is the curve slope around *x_0_*, *y_0_* is the lower limit, *a* is the difference between the upper and lower limits, and *x_0_* is the herbicide dose required for 50% plant growth reduction (GR_50_). WT: Wild type, IISS: *E. indica* population with mutation of Thr102Ile + Pro106Ser in EPSPS; LL: *E. indica* population with mutation of Pro106Leu in EPSPS; SS: *E. indica* population with mutation of Pro106Ser in EPSPS.
