# Supplementary material for: Potential Role of EPSPS Mutations in the Resistance of Eleusine indica to Glyphosate
Source: Int J Mol Sci. 2023 May 4;24(9):8250. doi: 10.3390/ijms24098250 (PMC10179075; doi:10.3390/ijms24098250)
Supplement: Supplementary file 1 [file ijms-24-08250-s001.zip › Supplementary files/Supplementary Table S2.docx]

Supplementary Table S2**.** Number of differentially expressed metabolites among groups.

| Treatment groups | Differentially metabolites | Down-regulated | Up-regulated |
| --- | --- | --- | --- |
| IISS vs WT | 281 | 223 | 58 |
| SS vs WT | 371 | 130 | 241 |
| LL vs WT | 309 | 225 | 84 |

Note: WT: Wild type, IISS: *E. indica* population with mutation of Thr102Ile + Pro106Ser in EPSPS; LL: *E. indica* population with mutation of Pro106Leu in EPSPS; SS: *E. indica* population with mutation of Pro106Ser in EPSPS. Differential metabolites were screened based on variable importance in projection (VIP) values > 1 and a false discovery rate < 0.05.
