# Supplementary material for: Potential Role of EPSPS Mutations in the Resistance of Eleusine indica to Glyphosate
Source: Int J Mol Sci. 2023 May 4;24(9):8250. doi: 10.3390/ijms24098250 (PMC10179075; doi:10.3390/ijms24098250)
Supplement: Supplementary file 1 [file ijms-24-08250-s001.zip › Supplementary files/Supplementary Table S3.docx]

Supplementary Table S3. Dose designed for dose-response assay.

| Formulation | Manufacturer | Populations | Dose (g a.e. ha^-1^) ^a^ |
| --- | --- | --- | --- |
| isopropylamine salt of glyphosate, 410 g a.e. L^-1^ | Bayer Crop Science | WT | 0, 112, 225, 450, **900**, 1800 |
|  |  | SS | 0, 225, 450, **900**, 1800, 3600 |
|  |  | LL | 0, 225, 450, **900**, 1800, 3600 |
|  |  | IISS | 0, **900**, 1800, 3600, 7200, 14400, 57600 |

^a^ The number in bold represents the recommend rate. For the experiment of excluding other non-target-site resistance mechanisms, control of spraying with water and spraying with only inhibitors was set under the glyphosate dose of ‘0’.
