# Supplementary material for: Potential Role of EPSPS Mutations in the Resistance of Eleusine indica to Glyphosate
Source: Int J Mol Sci. 2023 May 4;24(9):8250. doi: 10.3390/ijms24098250 (PMC10179075; doi:10.3390/ijms24098250)
Supplement: Supplementary file 1 [file ijms-24-08250-s001.zip › Supplementary files/Supplementary Table S4.docx]

Supplementary Table S4. Primers used in this study.

| Gene | Foward primer (5´-3´) | Reverse primer (5´-3) |
| --- | --- | --- |
| *EPSPS* clone | 5’-cgcggatccCACACTCTCTCTCTCTC-3’ | 5’-cccaagcttTTAGTTCTTGACGAAAGTGC-3’ |
| qPCR for *EPSPS* gene | GCAATTTCCCCAGTGACGACC | GCAAAAGCCTCTATCTTCCCTGT |
| Reference gene (*ALS*) | GGTGGCAAGGTTAAGTTATCTGG | TCAACATAAGGGATGGAGATCAG |
