# Supplementary figures and images for: Potential Role of EPSPS Mutations in the Resistance of Eleusine indica to Glyphosate
Source: Int J Mol Sci. 2023 May 4;24(9):8250. doi: 10.3390/ijms24098250 (PMC10179075; doi:10.3390/ijms24098250)

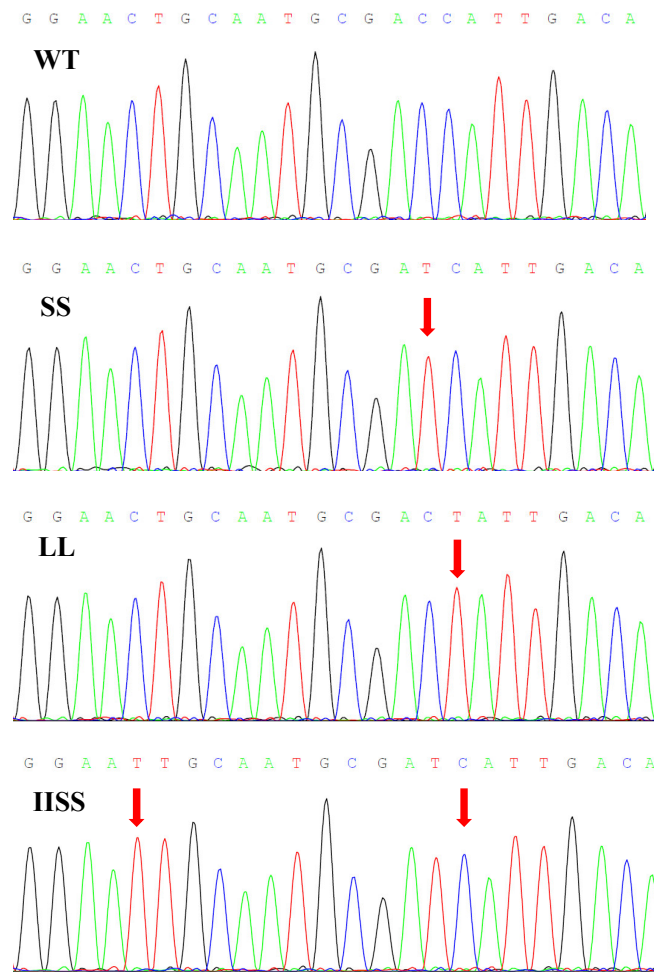

**Supplementary Figure S2.** Partial nucleotide sequence of EPSPS in four populations.

Supplement: Supplementary file 1 [file ijms-24-08250-s001.zip › ijms-2367490-supplementary/Supplementary Figure S2.pdf]

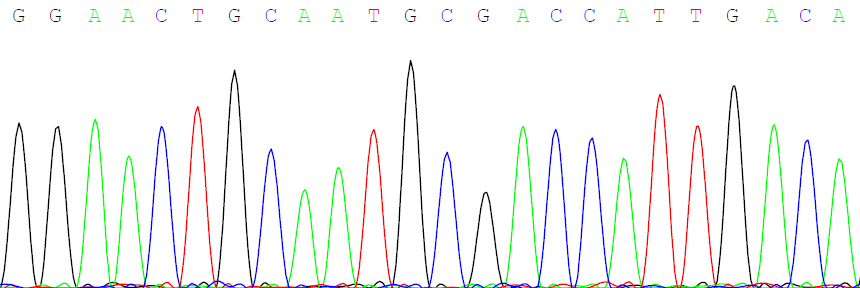

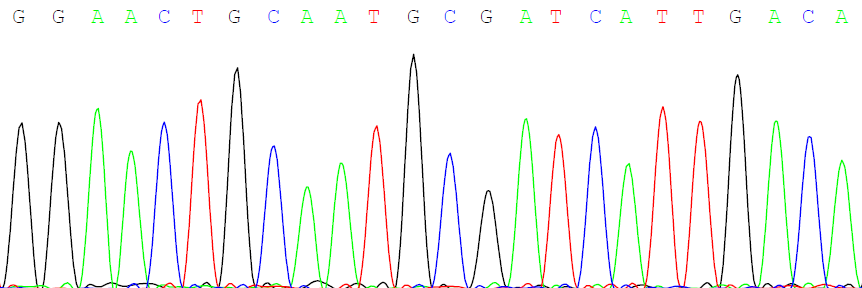


**WT**

**SS**


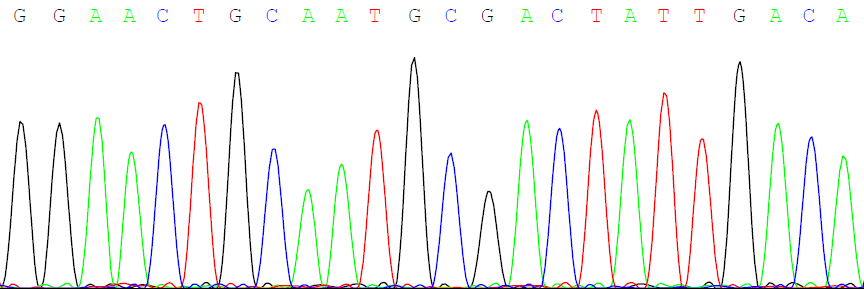


**LL**


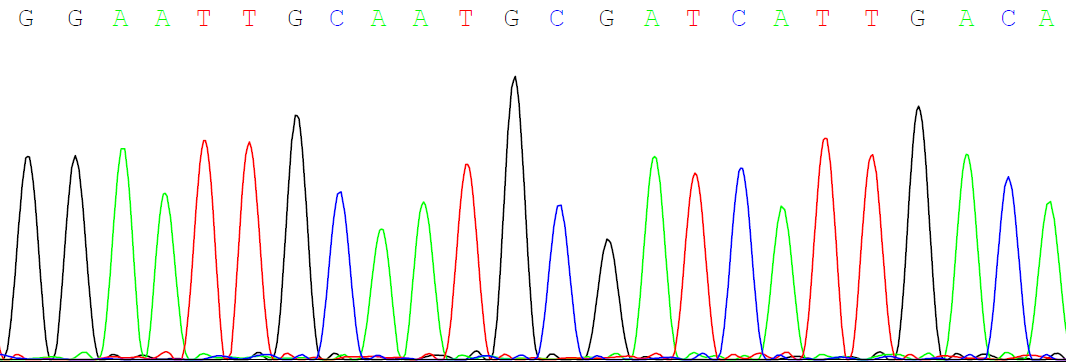


**IISS**

Supplementary Figure S2. Partial nucleotide sequence of EPSPS in four populations.

Supplement: Supplementary file 1 [file ijms-24-08250-s001.zip › Supplementary files/Supplementary Figure S2.docx]
